# Reduced antioxidant high-density lipoprotein function in heart failure with preserved ejection fraction

**DOI:** 10.1007/s00392-024-02583-3

**Published:** 2025-01-15

**Authors:** Benjamin Sasko, Theodoros Kelesidis, Sawa Kostin, Linda Scharow, Rhea Mueller, Monique Jaensch, Jan Wintrich, Martin Christ, Oliver Ritter, Christian Ukena, Nikolaos Pagonas

**Affiliations:** 1 Ruhr-University of Bochum, Medical Department II, Marien Hospital Herne, Bochum, Germany; 2 Department of Cardiology, Medical School Theodor Fontane, University Medical Center Brandenburg an Der Havel, Brandenburg an der Havel, Germany; 3 Division of Infectious Diseases and Geographic Medicine, Department of Medicine, University of Texas Southwestern Medical Center, Dallas, TX, USA; 4 Department of Cardiology, Medical School Theodor Fontane, University Hospital Ruppin-Brandenburg, Neuruppin, Germany; 5 Faculty of Health Sciences, Joint Faculty of the Brandenburg University of Technology Cottbus, Senftenberg, The (MHB) Theodor Fontane, and The University of Potsdam, Senftenberg, Germany; 6 Department of Cardiology, Knappschaftskrankenhaus Bottrop, Academic Teaching Hospital, University Duisburg-Essen, Bottrop, Germany; 7 Medical Department II, University Hospital Marien Hospital Herne, Hölkeskampring 40, 44625 Herne, Germany

**Keywords:** HDL function, Heart failure, Oxidative stress, Chronic inflammation, Lipid peroxidation

## Abstract

**Background:**

Heart failure (HF) is a heterogeneous clinical syndrome affecting a growing global population. Due to the high incidence of cardiovascular risk factors, a large proportion of the Western population is at risk for heart failure. Oxidative stress and inflammation play a crucial role in the pathophysiology of heart failure with preserved ejection fraction (HFpEF). While previous studies have demonstrated an association between dysfunctional HDL and heart failure, the specific link between oxidized HDL and HF remains unexplored.

**Methods:**

In this cross-sectional observational study, the antioxidant function of HDL was assessed in 366 patients with suspected heart failure. HFpEF assessment was conducted according to current guidelines. A validated cell-free biochemical assay was used to determine reduced HDL antioxidant function as assessed by increased HDL-lipid peroxide content (HDL_ox_), normalized by HDL-C levels and the mean value of a pooled serum control from healthy participants (nHDL_ox_; no units). Results were expressed as median with interquartile range (IQR).

**Results:**

Participants with HFpEF (*n* = 88) had 15% higher mean relative levels of nHDL_ox_ than those without heart failure (*n* = 180). Using a basic multivariate model adjusted for age, sex, eGFR and a full multivariate model (adjusted for diabetes, hypertension, atrial fibrillation, LDL cholesterol, hsCRP, and coronary artery disease), nHDL_ox_ was an independent predictor for HFpEF (*p* < 0.05). An increase in 1-SD in nHDL_ox_ was associated with a 67% increased risk for HFpEF if compared with participants without heart failure (*p* = 0.02).

**Conclusion:**

HDL antioxidant function is reduced in patients with HFpEF. Improving HDL function is a promising target for early heart failure treatment.

## Introduction

Heart failure (HF) is a significant health burden worldwide, with increasing numbers of hospitalizations, primarily caused by the aging and demographic development of the general population [1–4]. HF is a heterogeneous clinical syndrome consisting of clinical symptoms, such as dyspnea, pulmonary congestion, exercise intolerance, and edema, accompanied by objective signs of HF, including elevated plasma levels of B-type natriuretic peptide (BNP).

HF is caused by systolic or diastolic dysfunction or structural abnormalities of the heart. In the past decades, a wide heterogeneity regarding the classification of HF existed. However, all definitions had in common the left ventricular function (LVEF), which is considered to be the cornerstone for the classification of HF. According to the current HF guidelines both in Europe [5] and the United States [6], heart failure with preserved ejection fraction (HFpEF) is marked by clinical signs of HF despite a normal LVEF of ≥ 50%. Notably, this HF phenotype represents over 50% of all cases of HF and is driven by abnormal diastolic left ventricular filling resulting from impaired relaxation due to increased myocardial stiffness. As a consequence, ventricular–arterial uncoupling occurs, leading to increased LV filling pressure. This results in a backward failure with high left atrial pressure, increased pulmonary arterial pressure, pulmonary congestion, and right ventricular dysfunction.

Due to the high incidence of cardiovascular risk factors, a large proportion of the Western population is at risk for HF. Until now, current guidelines have not considered HDL-C as a possible contributor to HF. Very low and very high levels of high-density HDL-C are an important negative indicator of cardiovascular disease (CVD) and cardiovascular events. HDL function rather than absolute level (HDL-C) may be a more accurate indicator of CVD risk [7, 8] and prior studies confirm that CVD is strongly inversely associated with HDL function. While HDL is generally protective against the development and progression of CVD, in inflammation, HDL can be oxidized (HDL_ox_), thus becoming functionally impaired and increasing CVD risk [7, 9]. Independently of HDL levels, inflammation affects HDL by decreasing anti-inflammatory antioxidant factor levels and activity and by increasing associated pro-inflammatory proteins, lipid hydroperoxide content, and redox activity (HDL_ox_). Furthermore, functionally impaired HDL reduces cholesterol efflux potential and diminishes the ability of HDL to inhibit LDL oxidation [9].

Prior studies have shown an association between HDL_ox_ and inflammatory mediators that predict cardiovascular mortality, such as high-sensitivity C-reactive protein (hsCRP), IL-6 and serum amyloid A in inflammatory states [10, 11]. Furthermore, dysfunctional, pro-inflammatory HDL directly upregulates cytokine production, including TNF-α [12]. Thus, given that protective functions of HDL attenuate pro-oxidant, pro-inflammatory, and pro-thrombotic mechanisms, impaired HDL may be a common instigator for the development and progression of HF. However, the link between oxidized HDL and heart failure remains unexplored. Existing studies have demonstrated an association between dysfunctional HDL and heart failure [13–15], supporting our hypothesis.

In this cross-sectional observational study, we aim to assess HDL’s antioxidant function in patients with confirmed HFpEF using a cell-free fluorometric method. We hypothesize that a reduced antioxidant function of HDL is associated with the presence of HFpEF.

## Methods

### Study design

In this prospective cohort study, 366 consecutive patients presenting with suspected symptoms of heart failure (e.g. dyspnea, orthopnea, ankle swelling, reduced exercise tolerance, fatigue) as admission diagnosis underwent HF assessment between 2019 and 2021 at the University Hospital of Brandenburg. Patients were stratified according to the findings of transthoracic echocardiography and NT-proBNP measurement.

In addition to evaluating left ventricular (LV) function, the echocardiographic study aimed to identify objective evidence of cardiac functional and structural abnormalities. These abnormalities were defined by the presence of at least one of the following: diastolic dysfunction, left atrial enlargement, left ventricular hypertrophy, or signs of elevated pulmonary artery pressure. NT-proBNP cut-off levels to rule-in for heart failure were defined according to Bettencourt as follows [16]: (1) Age < 50: NT-proBNP > 450 pg/ml, (2) Age 50–75: NT-proBNP > 900 pg/ml, (3) Age > 75: NT-proBNP > 1800 pg/ml.

Based on the results of the HF assessment, two cohorts were built: (1.) Patients with HFpEF, defined as LVEF of ≥ 50%, echocardiographic evidence of cardiac functional and structural alterations and increased levels of NT-proBNP (HFpEF cohort, *n* = 88), and (2.) patients without HF, defined as LVEF of ≥ 50% with normal range NT-proBNP levels (No-HF cohort, *n* = 180).

Patients with an LVEF < 50% (*n* = 98) were excluded from this study. Further exclusion criteria were acute conditions that cause cardiac decompensation (e.g., hypertensive emergency, acute coronary syndrome, or pulmonary embolism), known cancer disease, acute infectious disease, known rheumatic disease, and age < 18 years, active COVID-19 infection.

If a study-independent indication was given, a study-independent coronary angiography was performed. Demographic data, current diagnoses, and medical history were recorded from the electronic data system or by patient interview.

All participants gave written informed consent. The Declaration of Helsinki approved the study by the local ethics committees of the Medical Association of Brandenburg (Nr. AS69bB/2016).

### Biomarker and laboratory assessment

Due to the complexity of the HDL particles, the measurement of HDL function has been difficult for humans to study [17]. We have developed a cell-free fluorometric method that measures HDL-associated lipid peroxide content (HDL_ox_) and offers a reproducible and rapid method of determining HDL function [18, 19]. Blood sampling was performed within 48 h after admission. The serum was prepared from blood samples and cryopreserved (−80 °C). Standard clinical assays in the central laboratory unit of the hospitals obtained laboratory parameters. The estimated glomerular filtration rate (eGFR) was calculated using the MDRD (Modification of Diet in Renal Disease) formula. High-sensitivity C-reactive protein (hs-CRP) was measured using the Roche Tina-Quant CRP kit (Roche Diagnostics, Basel, Switzerland).

### Assessment of lipid peroxide content of HDL (HDL_ox_)

HDL_ox_ was quantified in serum using a previously validated fluorometric biochemical cell-free assay that measures HDL lipid peroxide content based on the oxidation of the fluoro-chrome Amplex Red [18, 19].

First, serum was depleted from apolipoprotein B (ApoB) by polyethylene glycol (PEG) precipitation. 50 μl of ApoB-depleted serum was then added to wells of a 96-well plate in duplicate, followed by the addition of 0.075 units per well of horseradish peroxidase (HRP) and 50 μM Amplex Red reagent for a total volume of up to 100 μl. HRP catalyzes the reaction of Amplex Red to resorufin in combination with endogenous peroxides. After 1 h, the fluorescence of resorufin at 535/590 nm wavelengths was determined using a Spark 10 M microplate reader (Tecan, Austria). ApoB-depleted sera from 10 healthy volunteers (not study participants) were pooled and used as experimental control in each plate to standardize the assay and minimize experimental variability. Mean fluorescence from each sample was normalized by the mean fluorescent readout of the pooled control and HDL-C using the following calculation: “normalized” oxidized HDL (nHDL_ox_) = [HDL_ox__sample × 47 (mg/dl)]/[HDL_ox__control × HDL-C sample (mg/dl)], where 47 mg/dl represents HDL-C of the pooled serum control. Samples were analyzed after the recruitment of the last patient. The intra-assay coefficient of variation (CV) was 6.7%. The inter-assay CV was 3.7%. All laboratory measurements were performed in Brandenburg, Germany.

### Echocardiography studies

Transthoracic echocardiography was standardized and performed in each patient during the hospital admission using parasternal long and short axis, apical four and two chamber view (Philips Epiq 7, Philips, Amsterdam, the Netherlands). The assessment included the measurement of septum size, left atrial area, diastolic function with pw-/tissue Doppler technique, systolic pulmonary artery pressure, and the LVEF using Simpsońs monoplane method. All measurements were digitally recorded according to the American College of Cardiology/European Society of Cardiology (ACC/ESC) guidelines. Digital post hoc analysis of each echocardiography was performed by either NP or BS.

### Statistical analysis

The distribution of data was checked using Shapiro–Wilk test. If the assumption of normal distribution was not rejected (*p*-value > 0.1), the comparison of the two groups was performed with the *t*-test (> 2 groups: *F* test). Mann–Whitney *U* test was applied if the standard distribution assumption was rejected (> 2 groups: Kruskal–Wallis test). Values are given as median with interquartile range. Chi-square test was used to compare the frequency of a categorical variable between independent groups. Pearson correlation coefficient was assessed for correlations between two continuous variables. Univariate logistic regression analysis evaluated the relationship between continuous and categorical variables with the presence of disease (HF vs no disease). Univariate linear regression analysis assessed the relationship between continuous and categorical variables with the constant variable nHDL_ox_. Multivariate analyses (logistic regression) were performed and adjusted for age, sex and eGFR in a base model, in addition to diabetes mellitus, hypertension, hsCRP, LDL and the presence of CAD in the multivariate model. An experienced statistician performed all statistical analyses using the SAS/STAT and GraphPad Prism software.

## Results

### Demographic characteristics of study participants

A total of 268 participants were enrolled in this study. Eighty-eight participants were included in the first cohort, including HFpEF patients. The second cohort consisted of 180 participants without HF. The characteristics of all study participants are shown in Table 1. Patients suffering from HF were older than patients without HF (*p* < 0.001). When comparing both cohorts, the proportion of female participants is higher in the HFpEF group than in the No-HF group (52% vs. 64%, *p* = 0.002). Patients with HFpEF were more frequently suffering from diabetes (*p* = 0.002), atrial fibrillation (p < 0.001), and impaired renal function (*p* < 0.001). Furthermore, HFpEF patients showed higher levels of CRP (*p* = 0.04) and hsCRP (*p* = 0.01) if compared with patients without HF.

The incidence of cardiovascular risk factors other than diabetes, CAD, and stroke did not differ between the groups.

### Echocardiographic study

Results regarding the findings of the echocardiography study can be found in Tables 2 and 3. There was no difference in LV function between patients with HFpEF (*n* = 88) and those without HF (*n* = 180). Patients with HFpEF suffered more frequently from structural and functional abnormalities, including diastolic dysfunction, LA enlargement, LV hypertrophy and increase systolic pulmonary artery pressure (all *p* < 0.001). All three grades of diastolic dysfunction were more common in the HFpEF group, with higher rates of *E*/*e*´ > 15 cm/s.

**Fig. 1:**
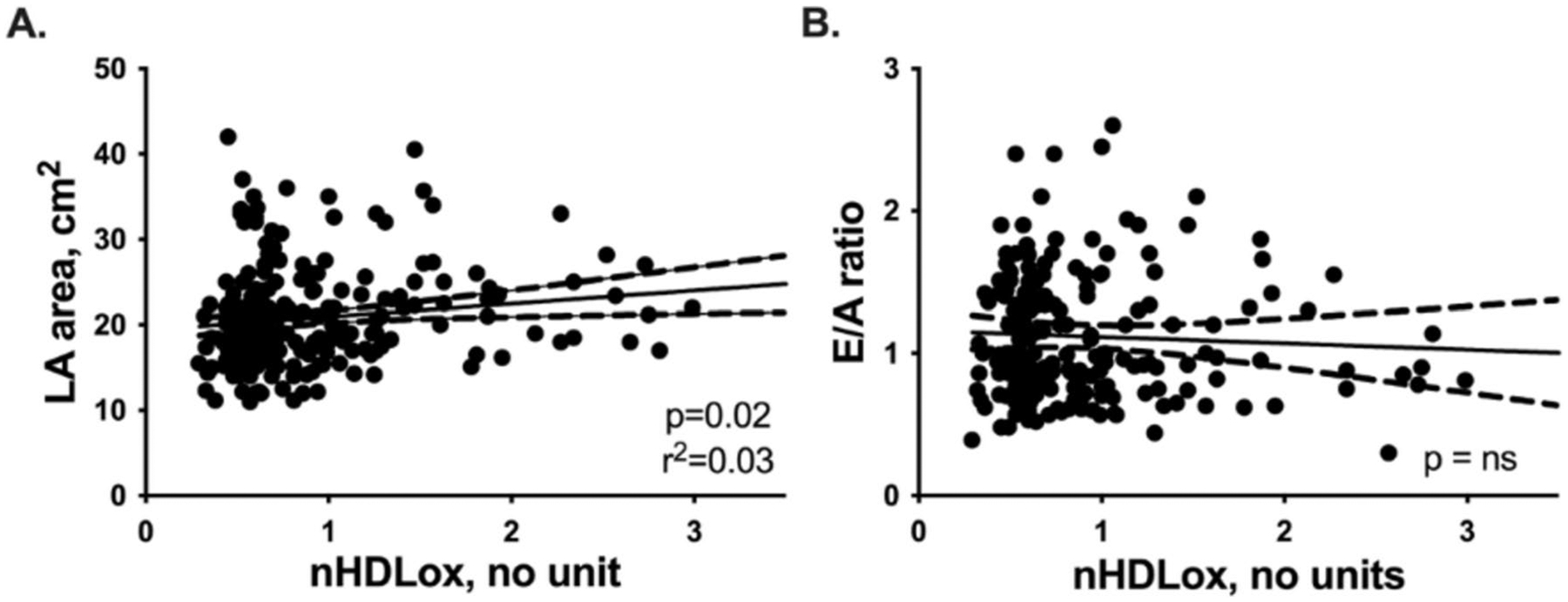
Correlation of nHDL_ox_ with LA area and E/A ration of the mitral valve. Linear regression analysis diagrams with 95% confidence bands of the best-fit lines. Spearman correlation coefficient (*r*^2^). Statistically significant values (*p* < 0.05) are shown in bold numbers

In linear regression analysis, we found an association between LA size and HDL_ox_ concentrations (*p* < 0.05, Fig. 1). Still, there was no correlation between the E/A ratio of the mitral valve as a measure of diastolic function and HDL_ox_ levels (Fig. 1). Furthermore, other correlations between oxidized HDL and echocardiographic findings associated with the HFpEF phenotype, as described in Table 2, could not be found (all *p* > 0.05, not included in the manuscript).

### Patients with HFpEF had a reduced antioxidant HDL function compared to participants without heart failure.

**Fig. 2:**
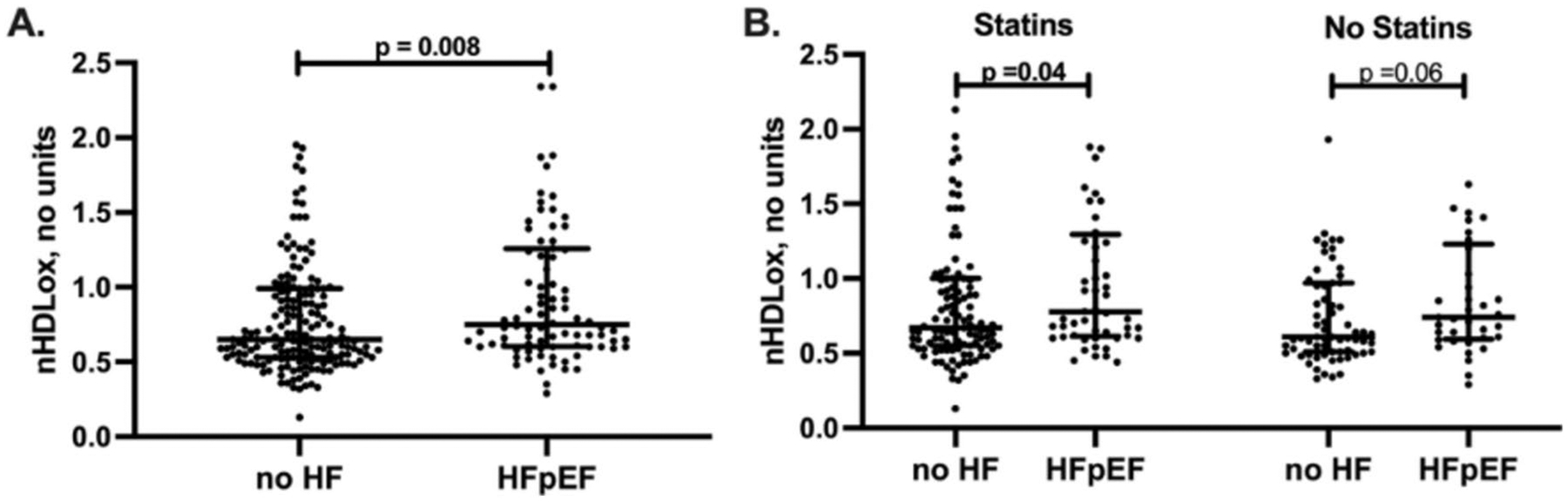
nHDL_ox_ in HFpEF versus no HF and in relation to statins. HDL antioxidant function (nHDL_ox_) was assessed in sera of patients with HFpEF and without HF by a biochemical assay as described in the method section. **A** nHDL_ox_ among participants with HFpEF and without HF. **B** nHDL_ox_ in relation to intake of statins or no intake. Kruskal–Wallis test was used. If significant, Mann–Whitney U test was used for pairwise comparison. Statistically significant values (*p* < 0.05) are shown in bold numbers

**Fig. 3:**
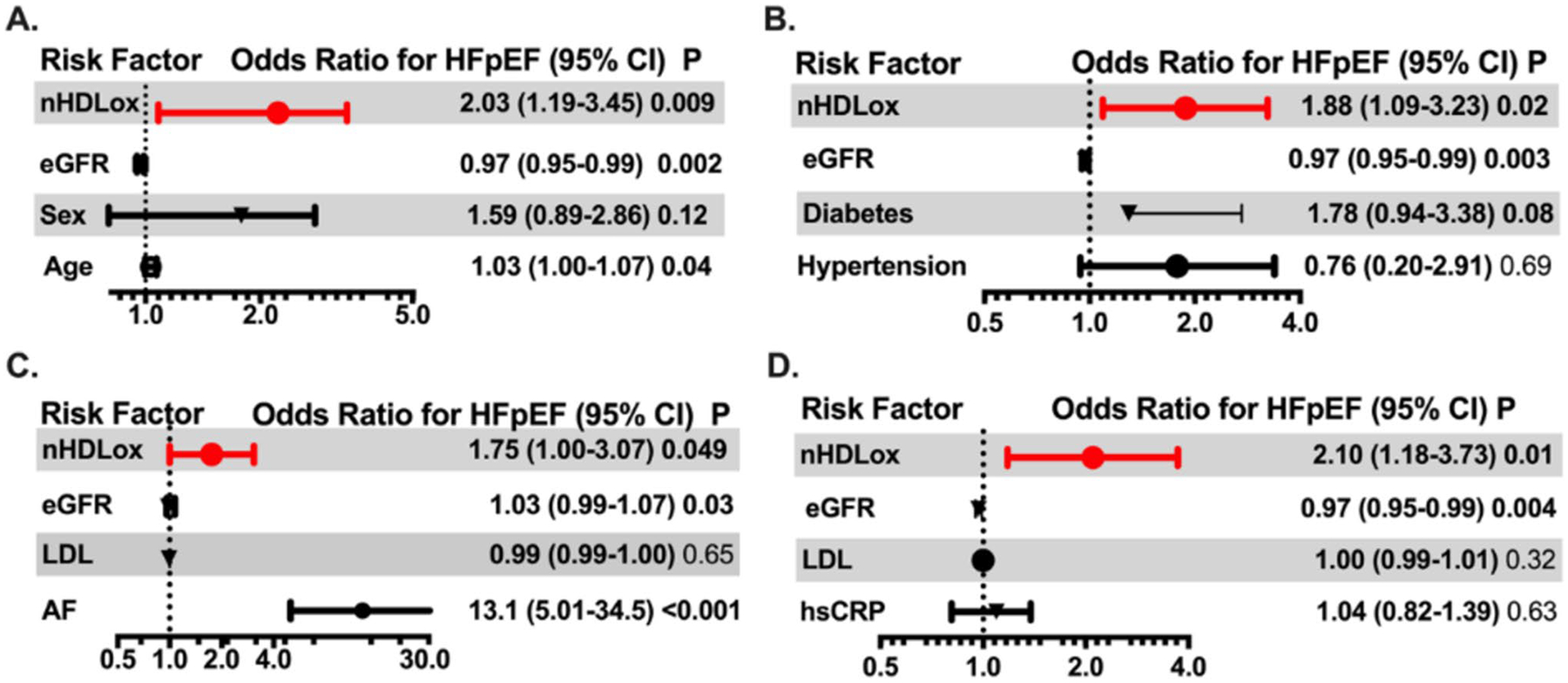
Associations of the antioxidant function of HDL with HFpEF. The antioxidant function of HDL (nHDL_ox_) was assessed in sera of patients with HFpEF or without heart failure (HF) by a biochemical assay as described in the method section. The associations of nHDL_ox_ with HFpEF (versus no HF) was assessed by logistic regression using a basic multivariate model adjusted for age, sex and eGFR (**A**) and different full multivariate models adjusted for selected risk factors and comorbidities (diabetes, hypertension, atrial fibrillation, LDL cholesterol and hsCRP (in addition to age, sex and eGFR, **B**–**D**). Odds ratios and 95% confidence intervals are shown. Odds ratios for continuous variables are per 1-SD increase

The 88 patients with HFpEF had a median of 15% higher nHDL_ox_ than the 180 patients without HF (*p* < 0.01, Table 1, Fig. 2). We determined the associations of nHDL_ox_ levels with the presence of HFpEF. In univariate logistic regression analysis of all study participants, an increase in 1-SD in nHDL_ox_ was associated with a 67% increased risk for HFpEF if compared with participants without heart failure (odds ratio 1.67, 95% confidence interval (CI), 1.07–2.6, *p* = 0.02). In multivariate analysis, nHDL_ox_ remained a significant risk factor for HFpEF after adjustment for age, sex and eGFR in a basic model (odds ratio 2.03, 95% confidence interval (CI), 1.19–3.45, *p* < 0.01, Fig. 3A). In the regression multivariate model, nHDL_ox_ remained a risk factor for the presence of HFpEF, independent of traditional risk factors (odds ratio 1.88, 95% confidence interval 1.09–3.23, *p* = 0.02, Fig. 3B) or the presence of CAD (odds ratio 2.01, 95% confidence interval 1.18–3.42, *p* = 0.01). In the multivariate analysis adjusted for hsCRP, a marker of the inflammatory milieu, HDL_ox_ remained significantly associated with HFpEF (Fig. 3D). Notably, atrial fibrillation was associated with the highest odds ratio for HFpEF (odds ratio 13.1, 95% confidence interval 5.01–34.5, *p* < 0.001, Fig. 3C), but nHDL_ox_ remained a risk factor in this model. Thus, a reduced antioxidant HDL function was consistently associated with HFpEF.

## Discussion

To our knowledge, this is the first study to date that has explored the association between oxidized HDL (HDL_ox_) as a measure of decreased HDL antioxidant function and the occurrence of heart failure. Our results consistently show that increased HDL_ox_ levels are associated with HFpEF in both univariate and multivariate analyses. Therefore, our main finding is that reduced antioxidant HDL function is present in heart failure with preserved ejection fraction.

Although the protective role of HDL against the development and progression of CVD is well established, less is known about the association of dysfunctional HDL and HF. Large RCTs that specifically tested the impact of HDL interventions on atherosclerotic cardiovascular disease did not show an impact on CVD risk through 90 days [20]. However, to our knowledge, no large RCT with an intervention that specifically targets HDL function has studied the impact on parameters of heart failure to reliably explore the biological and clinical relevance of impaired HDL function on pathogenesis of heart failure.

The functionality of HDL is closely linked to inflammation and oxidative stress. The pathogenesis of HFpEF is also characterized by systemic inflammation and oxidative stress [21]. HDL’s anti-inflammatory and antioxidant properties can potentially mitigate these pathological processes [22–24], thereby improving cardiac function. HDL can counteract these effects by reducing oxidative stress and inflammation, thus potentially improving endothelial function and reducing myocardial stiffness. It further facilitates cholesterol efflux and reverse cholesterol transport, which enhances the exchange of calcium ions (Ca^2+^) between cardiac muscle cells and the endoplasmic reticulum [25]. This process increases cardiac contractility and improves both systolic and diastolic function. Loss of antioxidant, anti-inflammatory, and endothelial protective properties of HDL could impact heart failure pathogenesis in several ways. Healthy HDL directly scavenges ROS and prevents LDL oxidation [26]. This antioxidative function is primarily mediated by the presence of antioxidant enzymes such as paraoxonase-1 (PON1). The oxidative modification of HDL reduces cholesterol efflux potential and diminishes HDL’s ability to inhibit LDL oxidation [27]. Oxidized HDL itself has reduced PON1 activity [28–30]. In animal models, oxidized HDL has also been found to induce cardiac fibrosis [31]. We have previously shown that HDL_ox_ is associated with atrial fibrillation [32], which is linked to structural cell damage and electro-physiologic changes in cardiomyocytes. In atrial myocytes, matrix metalloproteinases (MMPs) increase the production of matrix proteins in atrial tissue, leading to atrial fibrosis [33]. Conversely, HDL_ox_ induces cytotoxicity by triggering the release of MMP-9 and TNF-α from macrophages through NADPH oxidase-dependent mechanisms [34]. This results in the degradation of extracellular matrix components, leading to cardiac tissue remodeling and fibrosis [35]. HDL function is crucial in modulating fibrosis, and oxidized HDL appears to contribute to cardiac remodeling through various pathways. In summary, the combined effects of HDL_ox_-mediated endothelial dysfunction, inflammation, atherogenesis, and oxidative stress contribute to the deterioration of (micro) vascular blood flow, leading to reduced myocardial perfusion [36].

Aside from left atrial size, we did not find a linear association between HDL_ox_ levels and echocardiographic parameters typically associated with the HFpEF phenotype. This may be due to the relatively high incidence of echocardiographic evidence of diastolic dysfunction in the cohort without heart failure. This is consistent with previous studies showing increased oxidative stress and inflammation in heart failure patients [37]. Therefore, structural damage may be present but not necessarily linked to elevated oxidized HDL, unlike in clinically manifest heart failure.

Our findings indicate higher rates of chronic kidney disease and elevated levels of HDL_ox_ in HFpEF patients. This supports the concept of cardiorenal syndrome, which describes the bidirectional interaction between the heart and kidneys, where damage to one organ frequently leads to dysfunction in the other. This syndrome is characterized by increased oxidative stress and inflammation, which contribute to the formation of dysfunctional HDL [38], further exacerbating cardiovascular complications. However, our results from the multivariate analysis show that HDL_ox_ is elevated in the presence of heart failure, independent of kidney function. It can be summarized that impaired kidney function and impaired HDL function coexist in this group even though our data do not demonstrate a dependency between the two observations. Similar findings and conclusion can be drawn in the presence of diabetes mellitus, which also impairs HDL function [39]. Our results from the multivariate analysis, however, demonstrate that HDL_ox_ levels are increased in the presence of heart failure, independent of a diabetes mellitus.

Clinical and biological importance of HDL-associated lipid peroxide content remains unclear. The measure of HDL-associated lipid peroxide content has been validated to be biologically and clinically relevant in multiple independent human diseases including atherosclerotic cardiovascular disease [40, 41], atrial fibrillation [32], diabetes [42], metabolism [43], exercise physiology in hypertension [44] and chronic infections like HIV [11, 18, 19, 41, 45] and HDLV infection [46]. In one study that determined multiple independent measures of HDL function within the same person over time at baseline and 1 year following vertical sleeve gastrectomy (VSG), determination of HDL lipid peroxide content outperformed other assays of HDL function such as HDL cholesterol efflux [43]. However, further large longitudinal studies that will determine independent measures of HDL function in relation to clinical outcomes of heart failure and cardiovascular disease are needed.

The focus of this manuscript was the relationship of HDL antioxidant function with heart failure. We did not focus on the associations of HDL function with systemic inflammation, but in the multivariate analysis adjusted for hsCRP as a marker of the inflammatory milieu, HDL_ox_ still is significantly associated with HFpEF. We have previously shown that increased HDL_ox_ is linked to reduced anti-inflammatory function of HDL and increased systemic inflammation and immune dysfunction [11, 18, 19, 41, 45, 47, 48]. Thus, although we did not measure other parameters of HDL dysfunction and biomarkers of inflammation (due to limited biospecimens and resources), determination of HDL_ox_ may be relevant for pathogenesis of heart failure where both inflammation and oxidative stress are involved. Notably, HDL is linked to key pro-inflammatory molecular signatures that predict cardiovascular mortality such as serum amyloid A [10].

## Limitations

Our observational study does not prove causality and may be subject to unknown confounders, such as the use of various anti-arrhythmic, anti-coagulation, and anti-hypertensive medications, which may impact HDL function. Limitations of the analysis include lack of follow-up and lack of a predefined design. We did not determine other independent measures of HDL function, such as cholesterol efflux capacity. Given our large sample size and the known limitations of HDL function assays, including lack of standardization and significant heterogeneity about types of cells and type of readout reported, we utilized a well-standardized biochemical assay of HDL function with the inclusion of robust experimental controls. Furthermore, an impaired renal function may increase NT-proBNP levels, thus possibly confounding the diagnosis of HFpEF.

In summary, we found increased levels of oxidized HDL in HFpEF patients. Dysfunctional HDL_ox_ alters several key instigators in the pathogenesis of HFpEF, including oxidative stress, inflammation, apoptosis [24], NO production [49] and coronary microvascular function [21]. Thus, it is plausible to hypothesize that the oxidation of HDL significantly contributes to the development of HFpEF and improving HDL function is a promising target for early heart failure treatment. However, further studies are needed, focusing on the mechanistic impact of HDL_ox_ on the cardiomyocyte and endothelial function.

## Figures and Tables

**Table 1:** Baseline characteristics of all participants

	no HF	HFpEF	*p*-value
*N*	**180**	**88**	
Age, years	66 (57–76)	76 (66–80)	< **0.001**
Male, no (%)	116 (64)	46 (52)	**0.002**
Hypertension, no (%)	162 (90)	84 (95)	0.12
Diabetes, no (%)	38 (21)	34 (39)	**0.002**
Current smoking, no (%)	35 (19)	12 (14)	0.24
Obesity, no (%)	77 (43)	32 (36)	0.3
Atrial fibrillation, no (%)	35 (20)	34 (39)	< **0.001**
Stroke, no (%)	13 (7)	8 (9)	0.59
CAD, no (%)	87 (48)	53 (60)	0.06
BMI, kg/m^2^	28 (25–32)	28 (25–31)	0.42
nHDL_ox_, no unit	0.65 (0.53–0.98)	0.75 (0.6–1.2)	< **0.01**
HDL, mg/dl	52 (42–63)	52 (39–61)	0.22
LDL, mg/dl	109 (84–143)	110 (87–137)	0.52
Cholesterol, mg/dl	177 (148–212)	186 (153–218)	0.21
Triglyceride, mg/dl	115 (88–177)	124 (88–168)	0.17
Lipoprotein a, mmol/l	12 (6–44)	12 (7–39)	0.34
HbAlc, mmol/mol	5.7 (5.4–6.0)	5.9 (5.5–6.7)	**0.02**
NTproBNP, pg/ml	110 (55–218)	1072 (617–2047)	< **0.001**
eGFR	82 (71–92)	65 (49–82)	< **0.001**
Albumin mg/g Creatinine	2 (2–8)	9 (2–47)	< **0.001**
CRP	1.8 (0.9–4.0)	3.1 (1.1–7.0)	**0.04**
hsCRP	0.13 (0.07–0.42)	0.3 (0.09–0.67)	**0.01**

A *p*-value < 0.05 is regarded as significant (in bold)

**Table 2:** Description of echocardiography findings

	no HF	HFpEF	*p*-value
*N*	**180**	**88**	
Diastolic dysfunction, no (%)	80 (44)	79 (90)	< **0.001**
Grade I, no (%)	71 (88)	52 (66)	< **0.01**
Grade II, no (%)	8 (10)	23 (29)	< **0.001**
Grade III, no (%)	1 (2)	4 (5)	**0.02**
LV hypertrophy, no (%)	58 (32)	53 (60)	< **0.001**
LA dilation, no (%)	61 (34)	58 (73)	< **0.001**
PAsys > 35 mmHg, no (%)	22 (12)	44 (50)	< **0.001**

A *p*-value < 0.05 is regarded as significant (in bold)

**Table 3:** Presence of structural and functional abnormalities as assessed by echocardiography findings

	No HF	HFpEF	*p*-value
*N*	**180**	**88**	
LVEF, %	58 (53–62)	57 (53–61)	0.1
Diastolic dysfunction, no (%)	80 (44)	79 (90)	< **0.001**
Grade I, no (%)	71 (88)	52 (66)	< **0.01**
Grade II, no (%)	8 (10)	23 (29)	< **0.001**
Grade III, no (%)	1 (2)	4 (5)	**0.02**
*E/e*′	11 (8–14)	13 (11–15)	**0.01**
LV diameter end-diastolic, mm	49 (46–52)	50 (47–53)	0.15
LA area, mm^2^	18 (16–21)	23 (20–27)	< **0.001**
Interventricular septum, mm	11 (9–13)	13 (10–14.5)	< **0.001**
TAPSE, mm	17 (11–22)	24 (21–28)	< **0.01**
PAsys, mmHg	26 (24–30)	38 (33–48)	< **0.001**

A *p*-value < 0.05 is regarded as significant (in bold)

## Data Availability

The data that support the findings of this study are available from the corresponding author upon reasonable request.

## Abbreviations

HF: Heart failure
HFpEF: Heart failure with preserved ejection fraction
HDL: High-density lipoprotein
nHDL_ox_: Normalized HDL-lipid peroxide content
CAD: Coronary artery disease
AF: Atrial fibrillation
ACS: Acute coronary syndrome
CVD: Cardiovascular disease
BMI: Body mass index
LA: Left atrium
